# *N*,*N*-Bis(diphenyl­phosphan­yl)benzyl­amine

**DOI:** 10.1107/S1600536811000997

**Published:** 2011-01-12

**Authors:** Xu-Feng Liu, Wei-Hong Xu, Hui Li

**Affiliations:** a Department of Chemical Engineering, Ningbo University of Technology, Ningbo 315016, People’s Republic of China; b Department of Applied Chemistry, Yuncheng University, Yuncheng, Shanxi 044000, People’s Republic of China

## Abstract

In the title compound, C_31_H_27_NP_2_, the diphenyl­phosphanyl groups are staggered relative to the PNP backbone. The N atom is displaced by 0.219 (2) Å from the plane formed by the two P atoms and the methylene C atom. The angles around the N atom are 120.84 (16), 113.29 (16) and 120.57 (12)°, indicating that it exhibits a distorted trigonal–pyramidal geometry. There are no classical inter­molecular inter­actions.

## Related literature

For a related structure, see: Cloete *et al.* (2008 ▶).
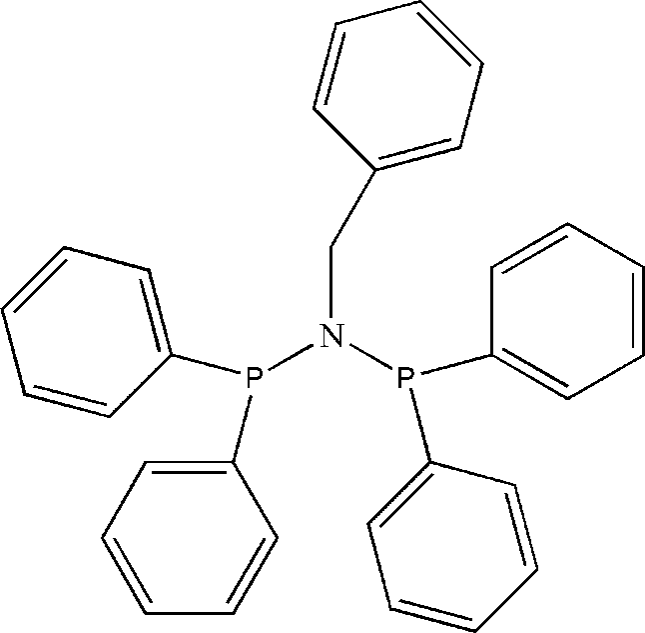


## Experimental

### 

#### Crystal data

C_31_H_27_NP_2_
*M* *_r_* = 475.48
Triclinic, 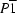

*a* = 10.381 (2) Å
*b* = 10.455 (2) Å
*c* = 13.239 (3) Å
α = 69.71 (3)°
β = 79.24 (3)°
γ = 70.21 (3)°
*V* = 1264.2 (4) Å^3^
*Z* = 2
Mo *K*α radiation
μ = 0.19 mm^−1^
*T* = 293 K
0.24 × 0.20 × 0.16 mm

#### Data collection

Rigaku Saturn diffractometer
Absorption correction: multi-scan (*CrystalClear*; Rigaku/MSC, 2005 ▶) *T*_min_ = 0.955, *T*_max_ = 0.970
11566 measured reflections
4453 independent reflections
3496 reflections with *I* > 2σ(*I*)
*R*_int_ = 0.040

#### Refinement

*R*[*F* ^2^ > 2σ(*F* ^2^)] = 0.059
*wR*(*F* ^2^) = 0.173
*S* = 1.04
4453 reflections
308 parameters
H-atom parameters constrained
Δρ_max_ = 0.92 e Å^−3^
Δρ_min_ = −0.34 e Å^−3^

### 

Data collection: *CrystalClear* (Rigaku/MSC, 2005 ▶); cell refinement: *CrystalClear*; data reduction: *CrystalClear*; program(s) used to solve structure: *SHELXS97* (Sheldrick, 2008 ▶); program(s) used to refine structure: *SHELXL97* (Sheldrick, 2008 ▶); molecular graphics: *SHELXTL* (Sheldrick, 2008 ▶); software used to prepare material for publication: *SHELXTL*.

## Supplementary Material

Crystal structure: contains datablocks global, I. DOI: 10.1107/S1600536811000997/pv2373sup1.cif

Structure factors: contains datablocks I. DOI: 10.1107/S1600536811000997/pv2373Isup2.hkl

Additional supplementary materials:  crystallographic information; 3D view; checkCIF report

## Figures and Tables

**Fig. 1.**
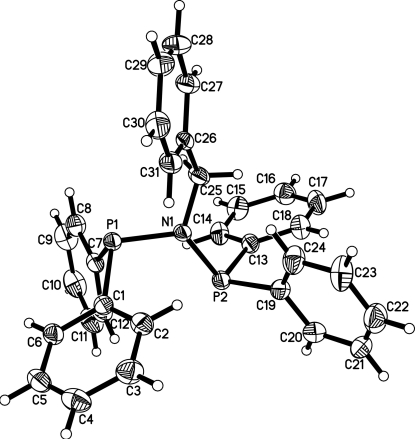
The molecular structure of the title compound. Displacement ellipsoids are drawn at the 30% probability level and H atoms are shown as small spheres of arbitrary radii.

**Fig. 2.**
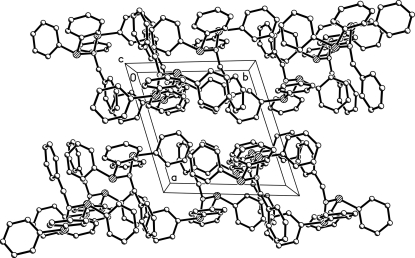
The crystal packing of the title compound.

## supplementary crystallographic information

### Comment

Diphosphine (PNP) ligands with different substituents have received considerable attention due to their potential applications in ethylene tetramerization catalyst systems (Cloete *et al.*, 2008).

In the title compound (Fig. 1), the diphenylphosphanyl groups are staggered relative to the P1/N1/P2 backbone. The N atom is displaced by 0.219 (2) Å from the plane formed by two P atoms and a C atom of the benzyl group. The angles around the N atom are 120.84 (16) °, 113.29 (16) °, and 120.57 (12) °, respectively, indicating that the nitrogen atom adopts a distorted trigonal-pyramidal geometry. The crystal structure is stabilized by van der Waals' interactions (Fig. 2).

### Experimental

To a solution of chlorodiphenylphosphine (1.8 ml, 10 mmol) and triethylamine (1.4 ml, 10 mmol) in CH_2_Cl_2_ (30 ml) was added dropwise benzylamine (0.55 ml, 5 mmol) at 273 K. The mixture was stirred at room temperature for 18 h. The solution was washed with NaOH solution. The organic phase was dried with MgSO_4_. Removal of solvent afforded a white solid of the title compound which was recrystallized from CH_2_Cl_2_ and ethanol (1:1) toafford colourless crystals.

### Refinement

All the H atoms were positioned geometrically (C—H = 0.93–0.97 Å) and refined as riding with *U*_iso_(H) = 1.2*U*_eq_(C) or 1.5*U*_eq_(methyl C).

### Crystal data

**Table:**

C_31_H_27_NP_2_	*Z* = 2
*M**_r_* = 475.48	*F*(000) = 500
Triclinic, *P*1	*D*_x_ = 1.249 Mg m^−^^3^
*a* = 10.381 (2) Å	Mo *K*α radiation, λ = 0.71073 Å
*b* = 10.455 (2) Å	Cell parameters from 3212 reflections
*c* = 13.239 (3) Å	θ = 2.1–27.9°
α = 69.71 (3)°	µ = 0.19 mm^−^^1^
β = 79.24 (3)°	*T* = 293 K
γ = 70.21 (3)°	Block, colourless
*V* = 1264.2 (4) Å^3^	0.24 × 0.20 × 0.16 mm

### Data collection

**Table:**

Rigaku Saturn diffractometer	4453 independent reflections
Radiation source: rotating anode	3496 reflections with *I* > 2σ(*I*)
confocal	*R*_int_ = 0.040
ω scans	θ_max_ = 25.0°, θ_min_ = 1.7°
Absorption correction: multi-scan (*CrystalClear*; Rigaku/MSC, 2005)	*h* = −12→12
*T*_min_ = 0.955, *T*_max_ = 0.970	*k* = −12→12
11566 measured reflections	*l* = −13→15

### Refinement

**Table:**

Refinement on *F*^2^	Secondary atom site location: difference Fourier map
Least-squares matrix: full	Hydrogen site location: inferred from neighbouring sites
*R*[*F*^2^ > 2σ(*F*^2^)] = 0.059	H-atom parameters constrained
*wR*(*F*^2^) = 0.173	*w* = 1/[σ^2^(*F*_o_^2^) + (0.1075*P*)^2^ + 0.1112*P*] where *P* = (*F*_o_^2^ + 2*F*_c_^2^)/3
*S* = 1.04	(Δ/σ)_max_ = 0.002
4453 reflections	Δρ_max_ = 0.92 e Å^−^^3^
308 parameters	Δρ_min_ = −0.34 e Å^−^^3^
0 restraints	Extinction correction: *SHELXL97* (Sheldrick, 2008), Fc^*^=kFc[1+0.001xFc^2^λ^3^/sin(2θ)]^-1/4^
Primary atom site location: structure-invariant direct methods	Extinction coefficient: 0.077 (9)

### Special details

**Table:**

Geometry. All e.s.d.'s (except the e.s.d. in the dihedral angle between two l.s. planes) are estimated using the full covariance matrix. The cell e.s.d.'s are taken into account individually in the estimation of e.s.d.'s in distances, angles and torsion angles; correlations between e.s.d.'s in cell parameters are only used when they are defined by crystal symmetry. An approximate (isotropic) treatment of cell e.s.d.'s is used for estimating e.s.d.'s involving l.s. planes.
Refinement. Refinement of *F*^2^ against ALL reflections. The weighted *R*-factor *wR* and goodness of fit *S* are based on *F*^2^, conventional *R*-factors *R* are based on *F*, with *F* set to zero for negative *F*^2^. The threshold expression of *F*^2^ > σ(*F*^2^) is used only for calculating *R*-factors(gt) *etc*. and is not relevant to the choice of reflections for refinement. *R*-factors based on *F*^2^ are statistically about twice as large as those based on *F*, and *R*- factors based on ALL data will be even larger.

### Fractional atomic coordinates and isotropic or equivalent isotropic displacement parameters (Å^2^)

**Table:**

	*x*	*y*	*z*	*U*_iso_*/*U*_eq_	
P1	0.09360 (6)	0.38539 (7)	0.11067 (5)	0.0374 (3)	
P2	0.26571 (7)	0.18718 (7)	0.29471 (5)	0.0419 (3)	
N1	0.1113 (2)	0.2572 (2)	0.23768 (15)	0.0370 (5)	
C1	0.2180 (2)	0.2936 (3)	0.02258 (19)	0.0387 (6)	
C2	0.2562 (3)	0.1463 (3)	0.0436 (2)	0.0482 (7)	
H2	0.2294	0.0896	0.1102	0.058*	
C3	0.3332 (3)	0.0827 (3)	−0.0330 (3)	0.0605 (8)	
H3	0.3582	−0.0159	−0.0176	0.073*	
C4	0.3727 (3)	0.1664 (4)	−0.1321 (3)	0.0649 (9)	
H4	0.4227	0.1244	−0.1845	0.078*	
C5	0.3384 (3)	0.3109 (4)	−0.1535 (2)	0.0555 (8)	
H5	0.3670	0.3666	−0.2199	0.067*	
C6	0.2620 (3)	0.3749 (3)	−0.0775 (2)	0.0466 (7)	
H6	0.2394	0.4734	−0.0932	0.056*	
C7	0.1735 (3)	0.5146 (3)	0.11555 (19)	0.0414 (6)	
C8	0.0838 (3)	0.6401 (3)	0.1334 (2)	0.0537 (7)	
H8	−0.0106	0.6565	0.1375	0.064*	
C9	0.1351 (4)	0.7407 (4)	0.1451 (3)	0.0715 (10)	
H9	0.0756	0.8239	0.1577	0.086*	
C10	0.2756 (4)	0.7153 (4)	0.1378 (3)	0.0735 (10)	
H10	0.3102	0.7815	0.1470	0.088*	
C11	0.3646 (4)	0.5951 (4)	0.1174 (2)	0.0664 (9)	
H11	0.4588	0.5808	0.1110	0.080*	
C12	0.3139 (3)	0.4948 (3)	0.1061 (2)	0.0501 (7)	
H12	0.3747	0.4130	0.0921	0.060*	
C13	0.2223 (3)	0.2333 (3)	0.4218 (2)	0.0432 (6)	
C14	0.2042 (3)	0.3744 (3)	0.4148 (2)	0.0550 (8)	
H14	0.2155	0.4404	0.3479	0.066*	
C15	0.1697 (4)	0.4166 (4)	0.5069 (3)	0.0643 (9)	
H15	0.1543	0.5118	0.5009	0.077*	
C16	0.1577 (3)	0.3205 (4)	0.6068 (3)	0.0627 (8)	
H16	0.1366	0.3498	0.6682	0.075*	
C17	0.1771 (4)	0.1816 (4)	0.6155 (2)	0.0682 (9)	
H17	0.1697	0.1157	0.6832	0.082*	
C18	0.2077 (3)	0.1378 (3)	0.5233 (2)	0.0590 (8)	
H18	0.2186	0.0432	0.5301	0.071*	
C19	0.2901 (3)	−0.0053 (3)	0.3464 (2)	0.0465 (7)	
C20	0.4192 (3)	−0.0896 (4)	0.3840 (2)	0.0628 (8)	
H20	0.4828	−0.0462	0.3865	0.075*	
C21	0.4526 (4)	−0.2377 (4)	0.4176 (3)	0.0760 (11)	
H21	0.5387	−0.2934	0.4417	0.091*	
C22	0.3580 (4)	−0.3011 (4)	0.4150 (3)	0.0749 (10)	
H22	0.3802	−0.4001	0.4371	0.090*	
C23	0.2312 (5)	−0.2198 (4)	0.3803 (3)	0.0780 (11)	
H23	0.1671	−0.2637	0.3797	0.094*	
C24	0.1982 (4)	−0.0737 (3)	0.3462 (3)	0.0627 (9)	
H24	0.1116	−0.0197	0.3224	0.075*	
C25	−0.0189 (3)	0.2644 (3)	0.3057 (2)	0.0476 (7)	
H25A	−0.0543	0.3578	0.3162	0.057*	
H25B	0.0001	0.1942	0.3760	0.057*	
C26	−0.1304 (3)	0.2389 (3)	0.2617 (2)	0.0412 (6)	
C27	−0.2631 (3)	0.2773 (4)	0.3079 (2)	0.0592 (8)	
H27	−0.2814	0.3202	0.3621	0.071*	
C28	−0.3695 (3)	0.2528 (4)	0.2748 (3)	0.0748 (10)	
H28	−0.4579	0.2781	0.3073	0.090*	
C29	−0.3446 (3)	0.1919 (4)	0.1947 (3)	0.0709 (10)	
H29	−0.4161	0.1771	0.1715	0.085*	
C30	−0.2131 (3)	0.1522 (3)	0.1481 (3)	0.0625 (8)	
H30	−0.1956	0.1098	0.0938	0.075*	
C31	−0.1065 (3)	0.1754 (3)	0.1822 (2)	0.0517 (7)	
H31	−0.0178	0.1475	0.1508	0.062*	

### Atomic displacement parameters (Å^2^)

**Table:**

	*U*^11^	*U*^22^	*U*^33^	*U*^12^	*U*^13^	*U*^23^
P1	0.0321 (4)	0.0439 (4)	0.0347 (4)	−0.0143 (3)	−0.0039 (3)	−0.0067 (3)
P2	0.0344 (4)	0.0498 (5)	0.0387 (4)	−0.0170 (3)	−0.0062 (3)	−0.0041 (3)
N1	0.0289 (10)	0.0492 (12)	0.0313 (11)	−0.0153 (9)	−0.0036 (8)	−0.0062 (9)
C1	0.0328 (13)	0.0487 (15)	0.0362 (14)	−0.0164 (11)	−0.0069 (10)	−0.0085 (12)
C2	0.0483 (16)	0.0526 (17)	0.0466 (16)	−0.0200 (13)	−0.0008 (13)	−0.0153 (13)
C3	0.061 (2)	0.0558 (18)	0.067 (2)	−0.0139 (15)	0.0009 (16)	−0.0282 (16)
C4	0.060 (2)	0.086 (2)	0.057 (2)	−0.0253 (18)	0.0100 (15)	−0.0365 (19)
C5	0.0526 (18)	0.074 (2)	0.0428 (17)	−0.0286 (15)	0.0044 (13)	−0.0161 (15)
C6	0.0404 (15)	0.0565 (17)	0.0413 (15)	−0.0193 (13)	−0.0038 (12)	−0.0080 (13)
C7	0.0451 (15)	0.0462 (15)	0.0332 (14)	−0.0199 (12)	−0.0020 (11)	−0.0072 (11)
C8	0.0608 (19)	0.0570 (18)	0.0441 (17)	−0.0183 (15)	−0.0058 (13)	−0.0146 (14)
C9	0.097 (3)	0.061 (2)	0.064 (2)	−0.0296 (19)	−0.0015 (19)	−0.0260 (17)
C10	0.105 (3)	0.075 (2)	0.061 (2)	−0.058 (2)	−0.004 (2)	−0.0172 (18)
C11	0.073 (2)	0.087 (2)	0.056 (2)	−0.051 (2)	−0.0017 (16)	−0.0172 (18)
C12	0.0466 (16)	0.0574 (17)	0.0483 (17)	−0.0249 (13)	−0.0024 (12)	−0.0102 (14)
C13	0.0388 (14)	0.0521 (16)	0.0376 (15)	−0.0189 (12)	−0.0091 (11)	−0.0040 (12)
C14	0.0646 (19)	0.0534 (18)	0.0467 (17)	−0.0223 (15)	−0.0119 (14)	−0.0064 (14)
C15	0.074 (2)	0.062 (2)	0.061 (2)	−0.0209 (17)	−0.0122 (17)	−0.0199 (17)
C16	0.067 (2)	0.075 (2)	0.0497 (19)	−0.0218 (17)	−0.0025 (15)	−0.0231 (17)
C17	0.083 (2)	0.069 (2)	0.0392 (18)	−0.0206 (18)	−0.0024 (16)	−0.0033 (16)
C18	0.073 (2)	0.0525 (17)	0.0500 (18)	−0.0209 (16)	−0.0067 (15)	−0.0102 (14)
C19	0.0424 (15)	0.0517 (16)	0.0385 (15)	−0.0087 (12)	−0.0050 (11)	−0.0099 (12)
C20	0.0447 (17)	0.067 (2)	0.062 (2)	−0.0108 (15)	0.0009 (14)	−0.0100 (16)
C21	0.061 (2)	0.065 (2)	0.059 (2)	0.0112 (18)	0.0028 (16)	−0.0004 (17)
C22	0.097 (3)	0.0476 (19)	0.067 (2)	−0.011 (2)	−0.003 (2)	−0.0126 (17)
C23	0.111 (3)	0.053 (2)	0.070 (2)	−0.030 (2)	−0.029 (2)	−0.0017 (17)
C24	0.071 (2)	0.0572 (19)	0.061 (2)	−0.0244 (16)	−0.0238 (16)	−0.0046 (15)
C25	0.0382 (15)	0.0668 (18)	0.0390 (15)	−0.0245 (13)	0.0023 (11)	−0.0118 (13)
C26	0.0329 (13)	0.0480 (15)	0.0365 (14)	−0.0191 (11)	−0.0025 (11)	0.0013 (12)
C27	0.0410 (16)	0.079 (2)	0.0530 (18)	−0.0223 (15)	0.0004 (13)	−0.0127 (16)
C28	0.0345 (17)	0.099 (3)	0.084 (3)	−0.0276 (17)	−0.0026 (16)	−0.013 (2)
C29	0.0446 (18)	0.089 (2)	0.078 (2)	−0.0345 (17)	−0.0196 (16)	−0.002 (2)
C30	0.061 (2)	0.071 (2)	0.0600 (19)	−0.0345 (16)	−0.0180 (15)	−0.0041 (16)
C31	0.0431 (16)	0.0624 (18)	0.0503 (17)	−0.0241 (14)	−0.0059 (13)	−0.0087 (14)

### Geometric parameters (Å, °)

**Table:**

P1—N1	1.744 (2)	C15—C16	1.369 (4)
P1—C1	1.823 (3)	C15—H15	0.9300
P1—C7	1.832 (3)	C16—C17	1.364 (5)
P2—N1	1.717 (2)	C16—H16	0.9300
P2—C19	1.829 (3)	C17—C18	1.393 (4)
P2—C13	1.843 (3)	C17—H17	0.9300
N1—C25	1.472 (3)	C18—H18	0.9300
C1—C2	1.392 (4)	C19—C24	1.374 (4)
C1—C6	1.394 (4)	C19—C20	1.402 (4)
C2—C3	1.382 (4)	C20—C21	1.390 (5)
C2—H2	0.9300	C20—H20	0.9300
C3—C4	1.380 (5)	C21—C22	1.369 (5)
C3—H3	0.9300	C21—H21	0.9300
C4—C5	1.366 (5)	C22—C23	1.365 (5)
C4—H4	0.9300	C22—H22	0.9300
C5—C6	1.377 (4)	C23—C24	1.371 (4)
C5—H5	0.9300	C23—H23	0.9300
C6—H6	0.9300	C24—H24	0.9300
C7—C12	1.389 (4)	C25—C26	1.522 (3)
C7—C8	1.397 (4)	C25—H25A	0.9700
C8—C9	1.391 (4)	C25—H25B	0.9700
C8—H8	0.9300	C26—C31	1.374 (4)
C9—C10	1.383 (5)	C26—C27	1.384 (4)
C9—H9	0.9300	C27—C28	1.389 (4)
C10—C11	1.365 (5)	C27—H27	0.9300
C10—H10	0.9300	C28—C29	1.360 (5)
C11—C12	1.382 (4)	C28—H28	0.9300
C11—H11	0.9300	C29—C30	1.377 (5)
C12—H12	0.9300	C29—H29	0.9300
C13—C18	1.387 (4)	C30—C31	1.390 (4)
C13—C14	1.395 (4)	C30—H30	0.9300
C14—C15	1.383 (4)	C31—H31	0.9300
C14—H14	0.9300		
			
N1—P1—C1	103.38 (11)	C14—C15—H15	119.5
N1—P1—C7	104.88 (11)	C17—C16—C15	119.5 (3)
C1—P1—C7	102.31 (12)	C17—C16—H16	120.2
N1—P2—C19	104.40 (12)	C15—C16—H16	120.2
N1—P2—C13	102.91 (11)	C16—C17—C18	120.3 (3)
C19—P2—C13	100.61 (12)	C16—C17—H17	119.9
C25—N1—P2	120.84 (16)	C18—C17—H17	119.9
C25—N1—P1	113.29 (16)	C13—C18—C17	120.9 (3)
P2—N1—P1	120.57 (12)	C13—C18—H18	119.6
C2—C1—C6	117.8 (2)	C17—C18—H18	119.6
C2—C1—P1	122.8 (2)	C24—C19—C20	117.9 (3)
C6—C1—P1	118.7 (2)	C24—C19—P2	126.5 (2)
C3—C2—C1	121.2 (3)	C20—C19—P2	115.5 (2)
C3—C2—H2	119.4	C21—C20—C19	120.4 (3)
C1—C2—H2	119.4	C21—C20—H20	119.8
C4—C3—C2	119.6 (3)	C19—C20—H20	119.8
C4—C3—H3	120.2	C22—C21—C20	119.6 (3)
C2—C3—H3	120.2	C22—C21—H21	120.2
C5—C4—C3	120.0 (3)	C20—C21—H21	120.2
C5—C4—H4	120.0	C23—C22—C21	120.4 (3)
C3—C4—H4	120.0	C23—C22—H22	119.8
C4—C5—C6	120.6 (3)	C21—C22—H22	119.8
C4—C5—H5	119.7	C22—C23—C24	120.2 (4)
C6—C5—H5	119.7	C22—C23—H23	119.9
C5—C6—C1	120.7 (3)	C24—C23—H23	119.9
C5—C6—H6	119.6	C23—C24—C19	121.5 (3)
C1—C6—H6	119.6	C23—C24—H24	119.3
C12—C7—C8	118.8 (3)	C19—C24—H24	119.3
C12—C7—P1	125.2 (2)	N1—C25—C26	115.6 (2)
C8—C7—P1	116.0 (2)	N1—C25—H25A	108.4
C9—C8—C7	120.2 (3)	C26—C25—H25A	108.4
C9—C8—H8	119.9	N1—C25—H25B	108.4
C7—C8—H8	119.9	C26—C25—H25B	108.4
C10—C9—C8	119.2 (3)	H25A—C25—H25B	107.4
C10—C9—H9	120.4	C31—C26—C27	118.1 (3)
C8—C9—H9	120.4	C31—C26—C25	124.1 (2)
C11—C10—C9	121.3 (3)	C27—C26—C25	117.7 (2)
C11—C10—H10	119.4	C26—C27—C28	121.0 (3)
C9—C10—H10	119.4	C26—C27—H27	119.5
C10—C11—C12	119.6 (3)	C28—C27—H27	119.5
C10—C11—H11	120.2	C29—C28—C27	120.1 (3)
C12—C11—H11	120.2	C29—C28—H28	119.9
C11—C12—C7	120.8 (3)	C27—C28—H28	119.9
C11—C12—H12	119.6	C28—C29—C30	119.7 (3)
C7—C12—H12	119.6	C28—C29—H29	120.1
C18—C13—C14	117.9 (3)	C30—C29—H29	120.1
C18—C13—P2	124.8 (2)	C29—C30—C31	120.1 (3)
C14—C13—P2	117.3 (2)	C29—C30—H30	120.0
C15—C14—C13	120.2 (3)	C31—C30—H30	120.0
C15—C14—H14	119.9	C26—C31—C30	120.9 (3)
C13—C14—H14	119.9	C26—C31—H31	119.6
C16—C15—C14	121.1 (3)	C30—C31—H31	119.6
C16—C15—H15	119.5		
			
C19—P2—N1—C25	73.0 (2)	N1—P2—C13—C14	−76.3 (2)
C13—P2—N1—C25	−31.7 (2)	C19—P2—C13—C14	176.1 (2)
C19—P2—N1—P1	−134.41 (14)	C18—C13—C14—C15	−1.5 (4)
C13—P2—N1—P1	120.88 (14)	P2—C13—C14—C15	179.1 (2)
C1—P1—N1—C25	−145.17 (17)	C13—C14—C15—C16	2.5 (5)
C7—P1—N1—C25	107.98 (18)	C14—C15—C16—C17	−1.5 (5)
C1—P1—N1—P2	60.34 (15)	C15—C16—C17—C18	−0.5 (5)
C7—P1—N1—P2	−46.52 (16)	C14—C13—C18—C17	−0.5 (4)
N1—P1—C1—C2	28.8 (2)	P2—C13—C18—C17	178.9 (2)
C7—P1—C1—C2	137.6 (2)	C16—C17—C18—C13	1.5 (5)
N1—P1—C1—C6	−160.77 (19)	N1—P2—C19—C24	−4.7 (3)
C7—P1—C1—C6	−52.0 (2)	C13—P2—C19—C24	101.7 (3)
C6—C1—C2—C3	−1.1 (4)	N1—P2—C19—C20	171.5 (2)
P1—C1—C2—C3	169.4 (2)	C13—P2—C19—C20	−82.1 (2)
C1—C2—C3—C4	−0.3 (4)	C24—C19—C20—C21	1.3 (4)
C2—C3—C4—C5	1.5 (5)	P2—C19—C20—C21	−175.2 (2)
C3—C4—C5—C6	−1.3 (5)	C19—C20—C21—C22	−0.8 (5)
C4—C5—C6—C1	0.0 (4)	C20—C21—C22—C23	−0.3 (5)
C2—C1—C6—C5	1.2 (4)	C21—C22—C23—C24	0.9 (6)
P1—C1—C6—C5	−169.7 (2)	C22—C23—C24—C19	−0.3 (5)
N1—P1—C7—C12	77.6 (2)	C20—C19—C24—C23	−0.8 (5)
C1—P1—C7—C12	−30.0 (2)	P2—C19—C24—C23	175.3 (3)
N1—P1—C7—C8	−100.4 (2)	P2—N1—C25—C26	−143.99 (19)
C1—P1—C7—C8	152.0 (2)	P1—N1—C25—C26	61.6 (3)
C12—C7—C8—C9	−2.1 (4)	N1—C25—C26—C31	17.6 (4)
P1—C7—C8—C9	176.0 (2)	N1—C25—C26—C27	−164.9 (2)
C7—C8—C9—C10	0.6 (5)	C31—C26—C27—C28	−0.2 (4)
C8—C9—C10—C11	1.2 (5)	C25—C26—C27—C28	−177.8 (3)
C9—C10—C11—C12	−1.5 (5)	C26—C27—C28—C29	−0.9 (5)
C10—C11—C12—C7	−0.1 (5)	C27—C28—C29—C30	1.2 (5)
C8—C7—C12—C11	1.9 (4)	C28—C29—C30—C31	−0.5 (5)
P1—C7—C12—C11	−176.1 (2)	C27—C26—C31—C30	0.9 (4)
N1—P2—C13—C18	104.4 (3)	C25—C26—C31—C30	178.3 (3)
C19—P2—C13—C18	−3.2 (3)	C29—C30—C31—C26	−0.5 (4)
